# Supplementary material for: Increased sugar valuation contributes to the evolutionary shift in egg-laying behavior of the fruit pest Drosophila suzukii
Source: PLoS Biol. 2023 Dec 11;21(12):e3002432. doi: 10.1371/journal.pbio.3002432 (PMC10735178; doi:10.1371/journal.pbio.3002432)
Supplement: Attachment — Submitted filename: Cavey_Response_to_Reviewers.docx [file pbio.3002432.s014.docx]

**In addition to the changes and corrections requested by the reviewers, we have made the following changes:**

- Inspired by the reviewers’ comments, we have exchanged the order of Figs 1 and 2. We believe this improves the overall readability of our results and the quality of our narrative.

- We have added one condition (small chambers) in the new Fig 2A (previously Fig 1A).

- We have increased the sample size of one condition (1 vs 2% glucose) in Fig 2B (previously Fig 1B).

- We have moved the results of the experiment 0.2 vs 1.6% sugar (previously Fig 2E) to new Fig 2F.

- We have removed the average ∆F/F_0_ traces (previously Fig 4C and S3D), as they are redundant with the heat maps and the bar graphs presented in the same figure.

- We have removed the analysis of GCaMP fluorescence decay (previously S3B Fig), since we did not observe a clear difference between species in the new stimulations with fructose and sucrose. As we do not know the potential mechanism for this phenomenon and currently cannot explore it further, we decided to remove this aspect for simplicity.

**Response to reviewers:**

Reviewers comments in blue

Our responses in black

**Reviewer #1:**

In the manuscript entitled "Increased sugar valuation in oviposition behavior of the fruit pest Drosophila suzukii", Cavey et al. investigate the behavioural and physiological basis of niche adaptation (via oviposition) to ripe fruit in D. suzukii.

Complementing previous studies, they show that sugar is a major driver for Dsuz choice to oviposit on ripe vs. fermented fruit which is preferred by D. melanogaster. By developing novel genetic tools in Dsuz, they can directly test the behavioural importance of sugar sensing neurons for oviposition via silencing experiments, and they are able to perform Calcium imaging experiments in sugar sensing gustatory receptor neurons.

Technical merit and experimental design: The manuscript is well written, nicely illustrated and the presented data supports the conclusion of the authors. Experiments are well designed, controlled and technically well done. The novel genetic tools introduced for Dsuz represent a major effort and will open exciting new research opportunities in the future.

Novelty and significance: One could argue that many of the described experiments overlap with a recent publication using the same experimental system [23]. However, I think the complementary genetic approach taken by the authors, the careful dissection of sugar induced oviposition and the emerging hypothesis of central brain changes merit publication in Plos Biology. Especially the potential role of central brain circuits in behavioural adaptation is very exciting and will be a promising avenue for future studies the authors are well positioned to perform. This observation is of interest not only to Drosophila researchers but other (neuro)scientist interested in brain function and evolution.

Prior to publication however, I would like the authors to consider a couple of points:

A)    Several recent studies using Calcium imaging in Drosophila peripheral sensory neurons have described ON and OFF responses upon stimulus presentation and removal (e.g. PMID 34197729 in sweet neurons, Curr Biol. 2021 Aug 23;31(16):3525-3537.e6. doi: 10.1016/j.cub.2021.06.005.

Mechanisms of lactic acid gustatory attraction in Drosophila ). Given the tools the authors have available, I would love to see if there are some species-specific temporal dynamics in the shown sugar responses that could impact central interpretation of peripheral signals.

This is indeed an interesting idea, but the rapid removal of the stimulus is not possible with the Ca2+-imaging protocol we are using. We could address this point in a follow-up study, using a different protocol.

B)    The authors nicely show the species separation between Dmel and Dsuz when offering ripe or fermented fruit (Figure 2D) and that Dsuz choice is mainly driven by sugar content. Given the chemical analysis in Sup. Fig. 1, I wonder if the authors could add acetic acid to the Ripe substrate and make Dmel prefer this combination over fermented. This experiment would allow to disentangle the relative role of acetic acid and sugars for both species. In Dsuz, even though they prefer oviposition of acid containing substrate (Fig. 3E), the sugar input seem to be more important (contrary to Dmel).

To investigate the relative value of acetic acid (AA) versus sugar, particularly in the context of the choice between a ripe or fermented substrate for oviposition, we performed the following additional experiments:

First, we gave the flies (wild type *D. melanogaste*r or *D. suzukii*) the choice between a sweet substrate (1.6% sugar) and an agar substrate supplemented with 1% AA (Fig. 1D). We observed opposite preferences: *D. melanogaster* chose the AA side whereas *D. suzukii* chose the sugar side, supporting the idea that *D. melanogaster* is “specialized” for fermentation cues (such as AA) and *D. suzukii* for sugar.

Second, as suggested by the reviewer, we supplemented the ripe strawberry substrate with AA, and opposed it to a fermented substrate (Fig. 1E). Adding 1% AA to the ripe substrate reduced the preference of *D. melanogaster* for the fermented substrate but did not completely reverse its preference to ripe. This manipulation also increased *D. suzukii*’s preference for the ripe substrate, suggesting that under these conditions, AA is not repellent to *D. suzukii*.

These results, presented in a revised version of Fig. 1, indicate that AA does indeed have a higher value relative to sugar for *D. melanogaster* than for *D. suzukii*. However, in the context of a ripe vs fermented substrate and under the conditions we used (specific 1% AA concentration used and fermentation protocol), AA does not appear to have a higher value for *D. melanogaster* than for *D. suzukii.* We believe that these experiments have reinforced the idea that changes in responses to sugar are particularly interesting and relevant to *D. suzukii’s* evolution, and have therefore decided to include them in our revised version.

Minor points:

Figure 1A: Could the authors plot the number of eggs per species laid? They mention several times in the manuscript that Dsuz lays eggs on agar only (and it is depicted in Fig. 1C) but it could help to show this once at the beginning of the manuscript

We now show the number of eggs laid on plain agar (and on sweet substrates) in Fig. S2 (formerly Fig. S1). These plots show the raw number of eggs laid in the experiments shown in Fig. 2A and Fig. 2E.

Figure 1C: Please add strain labels

We have done the requested changes.

Line 82: delete one "increase(d)"

We have corrected this.

Line 123 : "D. suzukii does not discriminate concentrations ≥ 1% glucose, whereas D. melanogaster always shows preference for the higher concentration": This phrasing is a a bit misleading as there is a preference in Dsuz at higher concentrations and it prefers 2.5% sugar vs. plain agar. Could the authors please rephrase this sentence?

We changed the sentence to (line 189-190): *“D. suzukii* does not discriminate between concentrations when both are higher than ~1% glucose [...]”

Line 181: "We therefore developed a controlled- fermentation protocol to produce a chemically-defined, sugar-free fermented substrate using industrial strawberry purée as a starting ripe substrate (S1A Fig) ": I recommend adding here that the authors analysed the chemical composition and this is actually shown in Fig. S1A

We have added the following sentence in the text (lines 91-92): “we measured the concentration of fermentation markers *(*sugar, ethanol and acetic acid) to assess the outcome of our fermentation reaction”.

We have also added the table in S1B Fig, which shows the concentration of these chemicals in the egg-laying substrates we used throughout the paper.

**Reviewer #2:**

The submitted manuscript delves into the effects of sugar in the oviposition substrate preference of two fruit fly species: Drosophila suzukii (Dsuz) and D. melanogaster (Dmel). While Dsuz lays its eggs on ripe fruit, Dmel prefers fermenting ones. Sugar appears to be a complicated cue in Dmel, as it can have a positive or negative effect depending on various factors. The authors have taken care to control as many variables as possible, resulting in an extensive set of experiments that can be somewhat challenging to summarize in a concise manner. In essence, the authors found that Dsuz and Dmel differ in their assessment of sugar when it comes to egg-laying. Dsuz is more sensitive to sugar than Dmel and prefers substrates with up to 1.6% sugar. However, Dsuz responds to lower sugar concentrations than Dmel, indicating that it may be more sensitive to sugar. Surprisingly, the authors found that Dsuz has fewer sugar-sensitive GRNs that are less sensitive to sugar.

General comments:

The paper is well-written, and the experiments presented are done to a high standard. The results are, however, quite confusing, and I'm not sure how much wiser I'm about this issue after having read this manuscript. There is an obvious need for more work here, and not only in Dsuz, but also in Dmel, to figure out how sugar affects egg-laying behavior. Despite the complexity, and sometimes even counterintuitive nature of their findings, the authors present their data clearly and thoughtfully, and also provide some reasonable arguments that explain some of the odder findings, which make this research noteworthy.

 Specific comments:

Line 59-62: Most Drosophila do not feed on rotting fruit. Drosophila (i.e. Sophophora and Drosophila) occupy a wide range of niches, which of these is the most common we do not know, but fruit is unlikely to be the most prevalent breeding substrate. As for utilizing ripe fruit, and its novelty within Drosoohilidae, again, this is not known.

We have added the following sentence (lines 43-44): “Natural breeding sites for fruit flies are not very well documented but they are known to lay their eggs on decaying organic material, presumably among other substrates”.

Line 169-171: Unclear. I don't understand what the authors mean. In fact this whole section is a bit hard to follow.

We have modified the text and added the following sentence to better explain why we think *D. suzukii* seems to have a weaker preference for sugar than *D. melanogaster* (lines 177-180): “However, we found that *D. suzukii* is naturally stimulated to lay eggs by plain agar, whereas *D. melanogaster* is not (S2A Fig and see below). This effectively reduces *D. suzukii*’s apparent preference for sugar in these assays, complicating the interpretation of the two-choice experiments.”

We have added the raw data for the two-choice assays (S2A Fig) to directly show that *D. suzukii* lays a significant number of eggs on the plain agar substrate.

We have changed the sentence to which the reviewer refers to (lines 240-243): “Taken together, our results suggest that the weaker preference of *D. suzukii* for sugar observed in specific experimental contexts must be interpreted with caution and does not rule out a role for this cue in biasing *D. suzukii*’s preference towards sugar-rich substrates in natural contexts.”

Figure 2E: no stats reported.

This figure panel is now Fig. 2F, and we have added the p-value of the statistical test.

Line 211ff: Wouldn't the Orco mutants previously generated by these authors been a good tool to use here? It would quite clearly demonstrate whether sugar is indeed the main driver of this behavior.

We have previously established in Karageorgi et al. Current Biol. 2017 (PMID: 28285999) that egg-laying in *D. suzuki*i can be elicited by the bouquet of a ripe strawberry odor, and that this is Orco-dependent.

In the same paper, we also ablated the antennae of *D. suzukii* females to produce anosmic flies and exposed them to ripe vs. fermented oviposition substrate. This ablation of the antennae had a minimal effect on the preference for the ripe substrate, suggesting that this preference is mainly driven by gustatory cues (or that gustatory and olfactory cues are somewhat redundant).

Based on these results, we believe that testing the preference of the *D. suzukii Orc*o mutant for ripe or fermented substrates would be minimally informative.

In addition, olfactory responses are likely to depend on IRs in addition to ORs (e.g.: acetic acid), so *Orco* mutants would not completely remove olfactory inputs.

Figure 3C: The PI of the Dsuz Gr64af-Gal4>UAS-Kir2.1 line is not all that different from parental Gr64-gal4 control. Is that really significant, if so, must be tough and go. Moreover, the PI (ca 0.25) is roughly about the same as the PIs seen in Figure 1A.

There is a day-to-day variability in the preference index between experiments, which is why the results presented in the same graph are performed in parallel at the same time. For this particular experiment, we tested in parallel (i.e., on the same days) the *D. suzukii* WT3 line, which showed the same PI for the ripe substrate as the Gal4 and UAS parental lines. We have added this data point to the figure panel (now Fig 3D) to further highlight the effect of silencing the Gr64af neurons on reducing the preference for sweet substrates.

Line 269: Does Gr64af-Gal4 really label all sugar neurons? Please rephrase.

It is difficult to be definitive here, but Gr64af-Gal4 is commonly used and presented in the literature as a ‘pan’ sweet GRN driver in *D. melanogaster.*

Our counts for the *DsuzGr64af-Gal4* line are consistent with Single Sensilla Recordings from Wang et al. 2022 (PMID: 36398882). To make this point clearer, we have added a table in Fig 3C comparing neuronal counts in the two species obtained by the two approaches (i.e.: Gal4 reporter vs electrophysiology). We believe that these data show very good agreement between the two methods and show the level of error that can be expected.

We have softened the sentence to (lines 286-289): “In conclusion, our *DsuzGr64af-Gal4* line appears to target the full complement of sugar-GRNs in *D. suzukii* and produces similar effects in behavioral assays to existing tools in *D. melanogaster*.”

Figure 3D: The PI of Dmel CS looks to be not significantly different from 0. And also different from the PI in Figure 2C.

There is a day-to-day variability in the PI value, particularly in the ripe vs fermented substrate tests. For the sake of simplicity, we have removed the *D. melanogaste*r CS data point from this graph, as it was of minimal informative value.

Figure 3E: But there is an effect at 0.1%. Any explanation?

This panel has been moved (unchanged) to S3B Fig.

There is indeed a reduction in the preference for 0.1% AA when the Gr64af neurons are silenced in *D. suzukii*. We can only speculate about the underlying reasons. For instance, we can’t rule out the possibility that Gr64af neurons play a role at low concentrations and not at higher ones (e.g.: Gr64af neurons could be sensitive to very low concentrations and other neurons would take over at higher concentrations). A somewhat similar situation has been described in *D. melanogaste*r, where distinct taste pathways detect acetic acid in different contexts and elicits opposite behaviors (Devineni et al. 2019, Acetic acid activates distinct taste pathways in *Drosophila* to elicit opposing, state-dependent feeding responses, PMID: 31205005).

Line 280ff: Again why not use the Orco-Gal4?

As explained above, the ablation of the antennae to produce anosmic flies has little effect on the egg-laying preference of *D. suzukii* when choosing between ripe and fermented substrates. In addition, using Orco-Gal4 to silence ORNs would not abolish IR-dependent perception of fermentation cues such as acetic acid so we would not expect to observe any phenotype in these experiments.

Line 336ff. This section is rather confusing.

We have rewritten and simplified this section, in particular replacing the complicated sentences relating GRN synaptic activity to behavior with (lines 373-375): “This would imply that low sugar inputs from the PNS are more potent in inducing oviposition in *D. suzukii* whereas higher inputs would be required in *D. melanogaster*.”

Figure 4F: Curious that the addition of sugar to ripe fruit overrides the innate attraction to fermentation volatiles. I think this observation warrants some sentences.

We have added the sentence (lines 388-390): “Sugar is therefore a weak positive oviposition cue for *D. melanogaster*, capable of overcoming the attraction by fermentation cues only when present at very high concentrations".

Line 411ff: But…Dmel likes sugar, the more the merrier it seems. How does that fit with Dmel having inhibitory sugar GRNs, whereas Dsuz, that do not like high sugar concentrations, lack these?

*D. suzukii* likes high sugar concentrations but does not need them to initiate oviposition. The idea that the two species have different subsets of functionally distinct sugar-GRNs is interesting but highly speculative, and we believe that we should not delve too deeply into possible scenarios. For example, we could imagine that 'activating' and 'inhibitory' GRNs compete to promote/inhibit oviposition and have different response dynamics to sugar concentrations. The 'inhibitory' neurons would prevail at low concentrations, while the 'activating' neurons would prevail at higher concentrations. The absence or reduced number of "inhibitory" neurons in *D. suzukii* would shift oviposition acceptance to lower concentrations.

**Reviewer #3:**

This paper by Cavey et al. addresses how different Drosophila species have evolved to occupy different ecological niches, a useful model for studying evolutionary adaptations more broadly. D. suzukii prefers to lay eggs on ripe fruit, which is high in sugar, whereas D. melanogaster prefers fermented fruit, which contains less sugar. Does sugar perception contribute to this behavioral difference? The authors make three major findings. First, sugar stimulates egg-laying more strongly and at lower concentrations in D. suzukii than in D. melanogaster. In other words, D. suzukii is more responsive to sugar, despite the fact that it shows lower sugar preference in a two-choice assay (due to high basal levels of egg-laying). Second, sugar perception is required for D. suzukii to show preference for ripe fruit puree. Third, despite D. suzukii's increased behavioral responsiveness to sugar, it has fewer sugar neurons than D. melanogaster and the responses of those neurons are weaker.

Thus, the increased behavioral response likely arises from increased sensitivity in the downstream circuit.

 The experiments are thorough and rigorous, and the results are nicely synthesized. I particularly appreciated how the authors developed a controlled paradigm for fruit fermentation and how they dissociated sugar stimulation of egg-laying from two-choice sugar preference. The development and use of genetic tools for neuronal imaging and manipulation in a non-model species is also commendable. I would support publication of this study as a Short Report, provided that my comments below can be addressed.

Major comments:

1) The title and several portions of the text refer to "increased sugar valuation" in D. suzukii. I do not agree with this terminology. "Value" and "valuation" are technical terms in certain fields of neuroscience that have a specific meaning, which I do not think is appropriate for this study where it seems like "value" simply refers to the ability of sugar to drive egg-laying behavior and choice.

The concept of 'relative-value' has been used in several contexts, including to study how animals, including flies, evaluate the nutritional 'quality' of particular foods to guide their feeding preferences and behavior (e.g. Abu et al. 2018, Communicating the nutritional value of sugar in Drosophila, PMID: 29507251). We, like others before us, have extended this concept of 'relative value' to examine how strongly a particular substrate induces oviposition relative to another (e.g. Chen et al. 2022, A functional division of Drosophila sweet taste neurons that is value-based and task-specific, PMID: 35031566; Reward foraging task and model-based analysis reveal how fruit flies learn value of available options, PMID: 33007023; Vijayan et al. 2023, A rise-to-threshold process for a relative-value decision, PMID: 37407812). We therefore believe that it is appropriate to use this concept to describe the relative preference of *Drosophila* species for a particular substrate.

 2) Based on Figure 2D, the sugar concentration is an important factor driving D. suzukii's preference for ripe fruit, but is NOT an important factor driving D. melanogaster's preference for fermenting fruit. The importance of sugar perception for D. suzukii was verified by Kir silencing of sugar GRNs. Was a similar experiment performed for D. melanogaster (silencing sugar GRNs while testing preference for ripe vs. fermented puree)? The expectation would be that there is not a significant effect. I think the fact that two species use different cues to choose between the same two substrates (and make opposing choices) is very interesting and could be emphasized more, along with a description of the other cues that could be involved (e.g. acetic acid, ethanol).

We thank the reviewer for this comment which has led us to perform additional experiments that strengthen the paper.

First, we have extended our analysis of chemical cues that may be relevant to oviposition decisions in ripe vs. fermented substrates by introducing new experiments with the fermentation marker acetic acid. We first found that *D. suzukii* and *D. melanogaster* do indeed appear to be “specialized” on different oviposition cues, namely sugar vs fermentation cues, in the experiment where we opposed sugar against acetic acid at concentrations found in ripe vs fermented substrates (Fig. 1D). However, we found that the oviposition response of both species to acetic acid is quite complex, depending on the concentration used as well as the experimental context, and we therefore believe that further exploration of this issue should be the subject of an independent study.

Second, as predicted by the reviewer, inhibition of sweet GRNs in *D. melanogaster* (Gr64af>Kir) does not affect fermented vs. ripe preference (Fig. 3E). As with *D. suzukii*, we verified that this manipulation does not affect preference for acetic acid (Fig. S3B), as this could have complicated the interpretation of these results. We therefore conclude that sugar perception is critical for oviposition decisions in *D. suzukii*, but not in *D. melanogaster*, reinforcing the idea that sugar has become a critical driver of oviposition behavior during the evolution of *D. suzukii*.

3) In Figure 4, was calcium imaging performed for other sugars besides glucose? Seeing similar results with other sugars such as sucrose and fructose would greatly strengthen the claim that neurons in D. suzukii show weaker responses, different temporal dynamics, and a flatter dose-response curve.

We performed additional calcium imaging experiments with fructose and sucrose and obtained results consistent with our glucose data, with overall weaker responses in *D. suzukii* than in *D. melanogaster*. We have included this new data in Figure 4B-C.

Although performing an interspecies comparison of calcium imaging data can be complicated for the technical reasons raised by the reviewer (see comment below), we feel that we have addressed this caveat and, in addition, our results are consistent with the electrophysiological recordings of Wang et al. 2022 (PMID: 36398882). Thus, two independent methods converge to support the idea that *D. suzukii* sugar-GRNs are not more sensitive than those of *D. melanogaster*, pointing to the central nervous system as the locus of evolutionary changes in oviposition decision-making processes.

 4) For the imaging data, I would be careful when comparing the strength of neuronal responses across species. Even within a species, neuronal responses can vary between driver lines. Ideally using deltaF/F should normalize for differences in transgene expression, but I don't think this always is completely true, e.g. if baseline values are close to the detection threshold or maximal response values are at a ceiling. I do appreciate that the authors didn't overstate this claim and focused on comparing the shape of the dose-response curves and the temporal dynamics.

We were also concerned about this issue and decided to address it experimentally by performing control stimulations with KCl to obtain a measure of the maximum calcium response detectable in each species. As explained in the paper, our results showed lower responses to KCl in *D. suzukii* (Fig. 4C), raising the possibility that experimental differences in imaging conditions (signal-to-noise ratio) or physiological differences between species reduce the amplitude of GCaMP fluorescence changes in *D. suzukii*. We therefore decided to normalize the sugar responses to the maximum detectable responses (i.e., KCl responses) for each species (Fig. S4B). We found that the amplitude of the glucose responses no longer differed between species, nor did the dynamics of the responses across concentrations. These results are consistent with the electrophysiological data of Wang et al. 2022 for glucose. We therefore decided not to discuss the dynamics of the glucose responses further.

In contrast, the normalized responses to fructose remained clearly smaller in *D. suzukii* than in *D. melanogaster* at 2.5% concentration, again consistent with electrophysiological data from Wang et al. 2022. As this type of interspecies comparison of calcium imaging has rarely been performed, we prefer to remain cautious in our interpretation and have not highlighted these species differences in the paper. Instead, we conclude that GRNs are not more sensitive in *D. suzukii* than in *D. melanogaster*.

Minor comments:

1) For readers who are familiar with previous studies investigating egg-laying aversion to sugar in D. melanogaster (e.g. Yang et al., 2008; Vijayan et al., 2022) it might be helpful to add a sentence mentioning how sugar can be either attractive or aversive for oviposition (and potentially why?).

There is indeed quite some confusion about the role of sugar in *D. melanogaster* oviposition preference. This stems in part from the differences in the set-up (chamber size), and the substrate composition used in the different studies. In several of them, including in Yang et al, 2008 and Vijayan et al., 2022, 2023, the sucrose and plain agar/agarose substrates are supplemented with ethanol and acetic acid (in different concentrations in these studies). We note, however, that we are not the only lab showing that *D. melanogaster* prefers sweet substrates (pure sugar mixed with agar or agarose) over plain agar (e.g., Wang et al., 2022).

We have explained this in the text (lines 167-170): “Since the oviposition response of *D. melanogaster* to sugar has been shown to be context-dependent and can range from sugar preference to sugar rejection [27,28], we tested different experimental conditions (chamber size and substrate composition).”

In addition, we have directly explored the effect of context on sugar acceptance/rejection in the two species. We found that chamber size - in the range that we tested - did not affect sugar preference of either species (Fig 2A). In contrast, substrate composition did, the presence of acetic acid and ethanol was sufficient to reverse sugar preference to aversion for both species (S2B Fig).

Although the underlying mechanisms are not understood, our results are consistent with most published data.

2) Reference 23 was published in eLife last year; please update the citation.

We have corrected this (now ref 22).

3) In Figure 1A, it would be helpful to add text labels to the schematics so it's clear where the oviposition substrates are and what the circular thing in the center is.

We have done this.

4) The text mentions that the lab-controlled fermented substrate, starting from strawberry puree, is "sugar-free". I was initially confused about whether this means no additional sugar has been added or whether the natural sugars were somehow removed. Based on later experiments I assume the former, but this should be clarified.

We explain in the Methods section (lines 544-547) that the ripe strawberry purée that we use is not sweetened. However, to enhance the fermentation reaction we supplemented the ripe purée (diluted 1:2 in Milliq water) with 2g glucose /100ml. The natural fruit sugars (glucose and fructose) present in the ripe strawberry purée plus the added glucose are consumed by the microorganisms during our fermentation.

5) In Figure 2C, it would be nice to show statistics to know which species/strains have a preference that's significantly different from zero (e.g. a one-sample t-test).

The statistics indicating the preference for one of the two substrates are indicated by open circles (not statistically different from 0) and closed circles (significantly different from 0). This is explained in the legends of Fig1A and we have added a legend on Fig 1C (top left) where the first non-significant preference data are seen.

6) Can the authors speculate on why D. suzukii has a lower number of sugar GRNs, since this is counterintuitive based on their increased oviposition response to sugar? Could it be related to any known differences in feeding behavior?

To our knowledge, the feeding behavior of *D. suzukii* has not been characterized. We believe that it would be speculative to suggest reasons for the observed reduction in GRN numbers, and we prefer not to do so due to space limitations. However, we note that our data for sugar GRNs are consistent with electrophysiological counts (Wang et al. 2022) and that a reduction in bitter GRNs has also been reported for D. suzukii (Dweck et al. 2021, PMID: 36398882).

7) On line 385, I don't think it's fair to characterize Wang et al.'s conclusion as saying "sugar was not an important cue for D. suzukii's oviposition decisions". They suggested that taste cues, including sugar, are less important than in D. melanogaster, but that is not the same as "not important".

We have changed this sentence to (line 424-425): “This observation suggests that sugar perception may not be critical for *D. suzukii’s* oviposition decisions [22]”.

8) On line 390 of the discussion, the authors state, "D. suzukii's overall weaker preference for sugar in two-choice assays is most likely due to a high basal egg-laying rate on plain agar". This is a very important point that could have been made more clearly in the results section.

We have added the raw data for the two-choice assays (S2A Fig) to directly show that *D. suzukii* lays a significant number of eggs on the plain agar substrate. We have also modified the text in the results section and added the following sentence to better explain why we think *D. suzukii* has a weaker preference for sugar than *D. melanogaster* (lines 177-180): “However, we found that *D. suzukii* is naturally stimulated to lay eggs by plain agar, whereas *D. melanogaster* is not (S2A Fig and see below). This effectively reduces *D. suzukii*’s apparent preference for sugar in these assays, complicating the interpretation of the two-choice experiments.”

**Reviewer #4:**

Certain Drosophilids can contribute to substantial agricultural loss, most prominently the Asian species Drosophila suzukii, which has rapidly expanded in Europe and Americas.

A striking difference in the ecology of D. suzukii is that these animals lay their eggs in ripe fruits as compared to rottening fruits in other Drosophila species. The neuronal and evolutionary context of how this preference emerged and is controlled remains largely unknown. One difference between the two food types - ripe versus fermented- is the content of sugars and the processing of these substances. In the current manuscript Cavey and colleagues investigate the relevance of different sugars, fresh fruits and fermented fruits for oviposition choice in D. suzukii.

They first show that compared to melanogaster, which prefers sugar D. suzukii showed a lower preference to sugars as compared to D. melanogaster (in a "choice assay"; however both species show preference for sugars (in a "non-choice assay"); The authors also show that D. suzukii is "unique" in ripe fruit preference among Drosophilds.

Since in D. melanogaster Gr64 genes are involved in sugar preference, the authors tested next if the Gr64 expressing neurons are also responsive to ripe fruit. They used behavioral and Calcium imaging experiments to corroborate this notion.

While genetic tools in D. melanogaster are highly advanced and abundant, the same is not yet true for other Drosophilids. The generation of GCaMP and other tools (UAS-KIR, Gr64-Gal4) used here are elegant and powerful. It is evident that this is a major achievement.

Overall, this is an elegant manuscript addressing a critical question in the largely unexplored biology of D. suzukii. I feel particularly the technical advances with this model is impacting.

There are a few points that I feel would be beneficial to be addressed.

- Above all, the preference of D. suzukii to ripe fruits as compared to decomposing fruits is among the best-known facts about this species; also making it an actual pest for certain types of ripe fruits. It seems intuitive that there must be some difference between the ripe and fermenting fruits, that are differentially "attractive" between D. suzukii and e.g. D. melanogaster. The authors here propose that perception of sugars to be such an aspect. The provided data indeed are in agreement to this notion, however much remains unclear how this sweet-perception difference might contribute to the difference in behavior/ecology. The only point where this is taken up to some degree is fig suppl 1.; in which some glucose, fructose, acetic acid and ethanol differences between ripe and fermenting fruits are measured.  I feel at the very least this should be more discussed as it provides a "real-world" measurement and provides a possible basis of why animals chose differently.

We have clarified this point by describing in more detail the measurements of sugar, acetic acid and ethanol concentrations in our substrates. We have added the sentence (lines 91-93): “We measured the concentration of fermentation markers (sugar, acetic acid and ethanol) to assess the outcome of our fermentation reaction and found that sugars were effectively depleted (S1A-B Fig).”

We have also added a table showing the final concentrations of these chemicals in our oviposition substrates (S1B Fig).

We have also performed additional experiments described below which we believe address this and the following concern.

Given the context it’s hard as a reader to feel that the context is to some degree not satisfying. Integrating behavioral experiments with acetic acid or ethanol and the combination with sugars might give at least partly explanations. I feel such experiments would provide some more context.

We performed these new experiments suggested by the reviewer to assess the contribution of different cues present in ripe vs fermented substrates to oviposition decisions of the two species.

First, we opposed sugar to the fermentation marker acetic acid and observed opposite preferences for the two species correlating with their opposite preferences for ripe vs fermented substrates (Fig 1D). This shows that acetic acid has a higher value relative to sugar for *D. melanogaster.*

Second, we added acetic acid to the ripe substrate in a two-choice assay against fermented (Fig 1E), which is basically the reciprocal to adding sugar to the fermented substrate (as we did previously in the experiment presented in Fig 1F). We found that under the conditions tested, acetic acid had a positive effect on the substrate preference of both species.

We have performed additional experiments, not presented in the paper, with different concentrations of acetic acid which show a complex and species-specific relationship between oviposition value and acetic acid concentration. We will investigate this phenomenon further in follow-up studies.

Taken together, our results support the idea that the two species are differentially tuned to specific chemicals; *D. melanogaster* is particularly responsive to fermentation cues, *D. suzukii* to sugars. We believe this provides a broader view of the different evolutionary changes that may have contributed to behavioral divergence, although the underlying mechanisms remain to be investigated.

- The reduced GCaMP response in D. suzukii could simply be due to GCaMP expression levels or activity of Gal4/UAS. Providing a positive response (e.g. high KCl or similar) would provide evidence that this is not an artifact.

We were also concerned about this issue and have decided to address it experimentally by performing control stimulations with 1M KCl to obtain a measure of the maximum calcium response detectable in each species. As explained in the paper, our results showed lower responses in *D. suzukii* (Fig 4C), raising the possibility that experimental differences in imaging conditions (signal-to-noise ratio) between species reduce the amplitude of GCaMP fluorescence changes in *D. suzukii.* However, we cannot exclude the possibility that there are differences in neuronal physiology between species.

We therefore decided to normalize the sugar responses to the maximum detectable responses (i.e.:  KCl responses) for each species (S4B Fig). We found that the amplitude of glucose responses and their dynamics over a range of concentrations were no longer different between species, but the amplitude of fructose responses were lower in *D. suzukii* at certain concentrations. As we observe species differences only for some sugars and at certain concentrations, and as these differences are consistent with published electrophysiological data (Wang et al. 2022, PMID: 36398882), we consider these data to be meaningful. However, we have chosen to remain cautious in our interpretation and have not highlighted these species differences in the paper.

- In ripe fruit preference also D. simulans and willistoni show preference for ripe fruit. I wonder if the authors have a view or explanation on this observation.

We do not have an explanation, several species show interesting phenotypes in this analysis and this requires further investigation, in particular using multiple wild type strains for each species.
